# Effectiveness of a Novel Device in the Reduction of Cesarean Deliveries

**DOI:** 10.1155/2013/173278

**Published:** 2013-09-01

**Authors:** Daniel A. Burns

**Affiliations:** Chief of Obstetrics, Niagara Falls Memorial Medical Center, 621 10th Street Hodge 3, Niagara Falls, NY 14301, USA

## Abstract

*Objective*. To test the hypothesis that the use of the HEM-AVERT Perianal Stabilizer will result in a reduction of cesarean births and shorter duration of second-stage labor. *Study Design*. In a prospective controlled trial, 102 women scheduled for vaginal delivery were randomized to either the HEM-AVERT investigational device or control group. Ninety eight (98) patients completed the study. A chi-square test was used to evaluate the difference in the number of cesarean deliveries between the investigational and control groups. Duration of second-stage labor was assessed as a secondary outcome. *Results*. Six (6) of the 50 patients in the investigational group (12%) failed to deliver vaginally and required cesarean delivery. Comparatively, 19 of the 48 control patients (39.6%) required cesarean delivery. Duration of second-stage labor was shorter in the investigational group, but the difference was not statistically significant. Results from 4 patients were excluded due to protocol violations. *Conclusion*. The HEM-AVERT device effectively reduced the incidence rate of cesarean deliveries in the investigational group when compared to women who delivered without use of the device. This trial is registered with ClinicalTrials.gov NCT01739543.

## 1. Introduction

The rate of cesarean delivery has risen steadily in the United States since 1996 and currently ranks as the most common surgical procedure performed on women with published estimates ranging from 1.3 to 1.4 million procedures annually [1]. Preliminary numbers released in the National Vital Statistics Reports (October 2012) indicated that the US cesarean rate in 2011 was 32.8%, unchanged from 2010 [2]. The growth in cesarean procedures crosses all demographic boundaries as it is inclusive of all age, racial, ethnic, and economic groups.

Attempts at trial of labor (TOL) declined during this same time period. Branch and Silver examined first-birth experiences and found the primary cesarean rate increased from 23.9% in 1990 to 27.1% in 2003 [3]. They noted that among women falling into the low-risk category (delivering at term with singleton vertex presentation), they encountered a 20% increase in cesarean deliveries during the same time period from 19.6% to 23.5%. Solheim et al. postulated that if the cesarean rate continues its ascent at the current rate, cesarean deliveries will account for 56% of all deliveries by 2020 [4]. 

Additionally, several studies have examined the cesarean rates among patients with active management of labor. Active management includes patient education, commitment to stay with the patient throughout the entire course of labor, aggressive use of oxytocin, and early amniotomy. In total, Branch and Silver examined 7 separate active management studies involving 6149 patients. The overall cesarean rate for the active management group was 13.3% compared to 14.8% for the routine care group. Their results suggest that some measure of reduction may be obtained with active management of labor, but the reduction is neither substantial nor consistent [3].

This is the first study designed to determine whether the application of perianal pressure can lower the cesarean birth rate. The objective of this study was to compare the efficacy of a novel device used during delivery, the HEM-AVERT Perianal Stabilizer (Plexus Biomedical, Oakland, TN, USA), to delivery without use of the device in women with singleton pregnancies scheduled for planned vaginal delivery. We hypothesized that use of the HEM-AVERT device would reduce the number of cesarean deliveries by increasing the patients' ability to push more effectively.

## 2. Materials and Methods

This was a prospective, randomized, clinical trial comparing the efficacy of the HEM-AVERT Perianal Stabilizer to a control group in scheduled singleton vaginal delivery. The HEM-AVERT Perianal Stabilizer is manufactured from medical grade polycarbonate and gauze and uses medical grade hook and loop fastener adhesive strips. All of these materials are commonly used in various medical devices. The subject device is a noninvasive medical device designed to provide counter-pressure to the anus and perianal region during delivery. It was approved by the FDA to help prevent the occurrence of external hemorrhoids originating during vaginal childbirth.

All patients were treated at the Niagara Falls Memorial Medical Center (Niagara Falls, NY, USA) between May 2012 and January 2013. The study met the institution's standards and guidelines and was considered a nonsignificant risk study of an approved device used per labeling provision for an identical patient population. The study protocol, informed consent, and oversight were reviewed and approved by the institution's Ethics Committee and the study was correspondingly registered with ClinicalTrials.gov (no. NCT01739543). 

All patients treated by the investigator during the course of the study were approached to determine eligibility. Women were allowed to participate in the study provided that the following inclusion criteria were met: patient was scheduled for a vaginal delivery, patient examination indicated that this would be a singleton birth, and the patient was willing and able to comply with the study plan as indicated by understanding and signing the patient's informed consent form. Patients were excluded from participation if any of the following criteria were encountered during the course of the study: patient's prenatal information indicated that it would not be a singleton birth; the patient was scheduled for an elective cesarean delivery; the patient was scheduled for vaginal delivery with anticipated complications (i.e., breech presentation). Patient consent was performed by a member of the research team while the patient was between 1 and 5 centimeters of cervical dilation.

Block randomization was achieved through a computer-generated randomization schedule using the SAS 9.2 process plan. The allocation employed a 1 : 1 ratio with variable block sizes of 4 and 6. Randomization selection occurred when the patient was between 5 and 8 centimeters of dilation.

Labor and delivery for both groups were managed in accordance with American Congress of Obstetricians and Gynecologists standards. Patients assigned to both groups began delivery in the normal low lithotomy position. Investigational patients had the HEM-AVERT device placed during the second stage of labor once proper dilation was achieved. Placement was performed as follows. The polycarbonate base was placed against the patient's anus. The two hook-and-loop fastener adhesive strips were then attached to the patient's buttocks and outer thighs. The natural tension created by the straps served to press the polycarbonate base against the anus and kept it in place during the remainder of delivery. All clinical staff members were trained on the proper placement of the device prior to patient enrollment.

Data collected included medical and pregnancy history, duration of second-stage labor, delivery method (e.g., vaginal, cesarean, assisted), complications, and demographic characteristics. Although not a primary outcome of this study, adverse events associated with the delivery were also collected.

A variety of statistical methods were used to analyze the available data. A Fischer's exact test was used for categorical variables. The Wilcoxon Rank-Sum test was utilized for numeric variables (e.g., weight and age), and a Cochran-Mantel-Naenszel test with modified ridit scores was used for ordered outcomes (number of previous cesarean deliveries and vaginal births). 

A number needed to treat (NNT) measurement was calculated for the device's ability to reduce the incidence rate of cesarean births. The NNT represents the average number of subjects needed to be treated with the investigational device to prevent one additional cesarean delivery when compared with the control. The lower the NNT, the more effective the investigational device is thought to be compared to the control. The NNT in this study was 4 with an approximate 95% confidence interval of 2.27 to 9.03. A NNT of 4 patients means that, for every 4 patients who gave birth while using the investigational device, 1 patient avoided a cesarean delivery with the device. This patient would have otherwise experienced a cesarean delivery without the device. For this type of low-risk medical device, an NNT of only 4 patients is very low and comparable to other known therapies. The NNT is calculated as 1 divided by the absolute reduction in the cesarean rates and rounded to the nearest whole number (i.e., 1/0.276 = 3.6 ~ rounds to 4 patients).

## 3. Results

A total of 102 women were enrolled from May 2012 to January 2013. Results from 4 patients were removed (1 investigational and 3 control) for protocol violations (i.e., patients were not properly consented). Of the remaining 98 patients, 50 women were assigned to the investigational arm and received the HEM-AVERT device, and 48 women were assigned to the control arm. All of the patients assigned to the investigational group were able to be fitted with the device per the manufacturer's instructions for use. None of the patients assigned to either group required operative delivery.

The treatment groups were comparable demographically with no significant variances found in terms of patient age, weight, or number of previous vaginal births. The number of previous cesarean births was similar between the groups as well, with the majority of patients having either zero or one previous cesarean birth, as shown in Table 1. 

The primary efficacy analysis was to evaluate the difference in number of cesarean deliveries between the investigational and control groups. Six (6) women assigned to the investigational group required cesarean delivery compared to 19 patients in the control group. The chi-square test result indicated that this difference was statistically significant and showed that patients using the HEM-AVERT device had a lower cesarean rate (12.0% for HEM-AVERT patients versus 39.6% for control patients, *P* = 0.0017). Statistical significance was also demonstrated in favor of the investigational patients when a subset of primiparous participants was examined (Table 2). 

Previous studies have reported that women who receive epidural analgesia may encounter more difficulty pushing, thus prolonging labor. Cheng et al. presented results from a retrospective cohort of 38,273 women who delivered with or without epidural analgesia. They reported that the length of labor was statistically significantly longer for women who received epidural analgesia among both nulliparous and multiparous women [5]. 

In the current study, 22 investigational patients chose not to receive epidurals and of those 2 (9.0%) delivered by cesarean. Twelve (12) control patients declined epidurals and again 2 patients delivered by cesarean (16.7%). Both groups had considerably lower cesarean rates compared to patients who received epidurals. As shown in Table 3, 14.3% of investigational patients and 47.2% of control patients had cesarean births when epidural analgesia was administered. The leading causes for the decision to convert to cesarean delivery in this study were fetal distress, stalled labor, and dystocia. 

Secondary analyses in the study included comparisons of duration of second-stage labor, length of hospital stay, and the number of adverse events encountered. Duration of second-stage labor data was collected on patients who delivered vaginally. A Wilcoxon Rank-Sum test assessing null hypothesis of no difference in median second-stage labor showed the difference to be nonsignificant (*P* = 0.2135). Overall, the mean duration of second-stage labor was 24.9 minutes for investigational patients and 40.8 minutes for control patients. 

The median length of hospital stay for the mother was 53.3 and 56.5 hours for the HEM-AVERT and control groups, respectively. A Wilcoxon Rank-Sum test assessing the null hypothesis of no difference in median length of stay revealed statistically significant differences between treatment groups (*P* = 0.0130). The median infant length of hospital stay was similar between the two groups (46.8 and 48.0 hours for HEM-AVERT and control groups, resp.) and not significantly different (*P* = 0.0687) at the 0.05 level.

No complications were recorded among the investigational patients. Seven (7) adverse events (AEs) were recorded in the control group (16.7%), with the most common AE being the occurrence of hemorrhoids (12.5%). The purpose of this study was not to assess the occurrence of hemorrhoids. Therefore, all patients (control and investigational) were not examined for this condition after delivery. Occurrences were only recorded upon the complaint of patients. One control patient experienced a serious adverse event (i.e., fetal bradycardia). A breakdown of reported AEs is provided in Table 4.

## 4. Discussion

Prior to implementation of this study, the HEM-AVERT device was used successfully at our facility for its indicated purpose—prevention of delivery-induced hemorrhoids. As usage continued, a trend toward a decline in cesarean deliveries was noticed. We speculated that a possible explanation for this trend was the result of the positive pressure applied by the HEM-AVERT device to perianal tissue during second-stage labor. The HEM-AVERT device also provides the patient with a tactile target to push against, particularly in cases where epidural analgesia is administered and patients fail to push effectively.

The latest statistics suggest that the rate of cesarean deliveries may have reached its peak at 32.9% in 2009. Records for the 2 years following have shown a steady rate of 32.8, a slight decline from 2009. This still equates to an average of 1.3 million cesarean deliveries being performed on an annual basis. Active labor management, patient education, and relaxation therapies have demonstrated inconsistent results and are limited by the commitment of each facility to actively support and train personnel on these measures.

The effect of epidural analgesia on duration of second-stage labor remains controversial. Results from the current study found that women who received epidurals had significantly longer labor. However, the cesarean rate among the investigational patients who received epidural analgesia remained lower than the latest reported national average (9.0% versus 32.8%). 

Although a significant reduction in second-stage labor could have been anticipated, we speculate that use of the device may play a labor augmentation role which results in more effective pushing and delays the incidence of stalled labor. The device may also help patients who are reluctant to push due to previous postpartum experiences involving delivery-induced hemorrhoids. Duration of progressive second-stage labor would therefore not be reduced by cesarean intervention.

## 5. Conclusion

We found that applying perianal pressure with the HEM-AVERT device reduced the overall chance of cesarean delivery by 69.7% at our facility. This equates to a 27.6 percentage point reduction in cesarean births between the investigational and control groups (39.6% for controls patients versus 12.0% for investigational patients). In this study, the device demonstrated itself to be an effective tool in reducing both the rate of cesarean births and the duration of second-stage labor. A larger study is needed to confirm the findings presented here. Additionally, given the small number of women with a prior history of cesarean deliveries enrolled in the current study, there may be merit in conducting a future study to examine vaginal births after cesarean (VBAC) rates between users of this investigational device and nonusers.

## Figures and Tables

**Table 1:** (Efforts to manage the cesarean delivery rate) baseline information by treatment. Efficacy population.

Summary	Treatment group		*P* value
	HEM-AVERT	Control	
Number of patients	50	48	
Weight (lbs)			
*N *	50	48	0.6188^1^
Mean (SD)	180.6 (44.46)	183.2 (43.79)	
Median	171.5	174.0	
Min, max	108, 305	122, 350	
Age			
*N *	50	48	0.7192^1^
Mean (SD)	25.0 (5.29)	25.0 (6.24)	
Median	26.0	24.0	
Min, max	14, 36	16, 41	
Previous births	30 (60.0%)	23 (47.9%)	0.3108^2^
Number of previous cesareans			
0	48 (96.0%)	46 (95.8%)	0.9669^3^
1	2 (4.0%)	2 (4.2%)	
Number of previous vaginal births			
0	20 (40.0%)	25 (52.1%)	0.3501^3^
1	15 (30.0%)	11 (22.9%)	
2	6 (12.0%)	4 (8.3%)	
3	6 (12.0%)	4 (8.3%)	
4	2 (4.0%)	2 (4.2%)	
5	1 (2.0%)	0	
6	0	2 (4.2%)	

Statistical Method:

^1^Wilcoxon-Rank Sum test.

^2^Fisher's Exact test.

^3^Cochran-Mantel-Haenszel test.

**Table 2:** (Efforts to manage the cesarean delivery rate) cesarean rate by treatment. Efficacy population.

Summary	HEM-AVERT	Control	Chi-square *P* value
Number of patients	50	48	
Cesarean deliveries	6 (12.0%)	19 (39.6%)	0.0017

Results for primiparous women			
Number of patients	20	25	
Cesarean deliveries	3 (15.0%)	15 (60.0%)	0.0022

**Table 3:** (Efforts to manage the cesarean delivery rate) cesarean rate by epidural status.

Summary	Treatment Group		Fisher's Exact Test *P* value
	HEM-AVERT	Control	
Number of patients	50	48	
Overall cesarean rate (for all patients)	6/50 (12.0%)	19/48 (39.6%)	0.0024
Cesarean rate for patients that received an epidural	4/28 (14.3%)	17/36 (47.2%)	0.0072
Cesarean rate for patients that did not receive an epidural	2/22 (9.1%)	2/12 (16.7%)	0.6015
Cesarean rate for primiparous patients	3/20 (15.0%)	15/25 (60.0%)	0.0027
Cesarean rate for primiparous patients that received an epidural	2/14 (14.3%)	14/23 (60.9%)	0.0073
Cesarean rate for primiparous patients that did not receive an epidural	1/6 (16.7%)	1/2 (50.0%)	0.4643

**Table 4:** (Efforts to manage the cesarean delivery rate) adverse events (AEs) reported by treatment group.

Summary	Treatment group		Fisher's Exact test *P* value
	HEM-AVERT	Control	
Number of patients	50	48	
Patients experiencing one or more AEs	0	7 (14.6%)	0.0053
Patients experiencing one or more major AEs	0	1 (2.1%)	0.4898
Adverse events by type			
Fetal bradycardia	0	1 (2.1%)	0.4898
Hemorrhoids	0	6 (12.5%)	0.0117
Right sulcus tear	0	1 (2.1%)	0.4898
